# Analyses of metastasis-associated genes in IDH wild-type glioma

**DOI:** 10.1186/s12885-020-07628-0

**Published:** 2020-11-16

**Authors:** Xiaozhi Li, Yutong Meng

**Affiliations:** 1 Department of Neurosurgery, Shengjing Hospital of China Medical University, Shenyang, China; 2 Department of Stomatology, Shengjing Hospital of China Medical University, No. 36 Sanhao Street, Shenyang, Liaoning Province 110004 People’s Republic of China

**Keywords:** Glioma, Metastasis, Prognosis, Invasion, Single-cell sequencing

## Abstract

**Background:**

Glioma is the most common malignant tumor of the brain. The existence of metastatic tumor cells is an important cause of recurrence even after radical glioma resection.

**Methods:**

Single-cell sequencing data and high-throughput data were downloaded from GEO database and TCGA/CGGA database. By means of PCA and tSNE clustering methods, metastasis-associated genes in glioma were identified. GSEA explored possible biological functions that these metastasis-associated genes may participate in. Univariate and multivariate Cox regression were used to construct a prognostic model.

**Results:**

Glioma metastatic cells and metastasis-associated genes were identified. The prognostic model based on metastasis-associated genes had good sensitivity and specificity for the prognosis of glioma. These genes may be involved in signal pathways such as cellular protein catabolic process, p53 signaling pathway, transcriptional misregulation in cancer and JAK-STAT signaling pathway.

**Conclusion:**

This study explored glioma metastasis-associated genes through single-cell sequencing data mining, and aimed to identify prognostic metastasis-associated signatures for glioma and may provide potential targets for further cancer research.

## Background

Glioma is the most common malignant tumor in the brain. In recent years, research on glioma especially on the molecular mechanisms has been increasing. In 2016, the World Health Organization first applied histology and molecular classification to define central nervous system tumors simultaneously [1]. Although with the continuous improvement of glioma diagnosis and treatment technology, the 5-year survival rate of patients with grade IV glioma is less than 5% [2], and the median survival time is about 12–15 months [3].

Glioma is composed of the tumor core and metastatic tumor cells. The metastatic tumor cells can be located a few centimeters away from the tumor core area. The existence of metastatic tumor cells is an important cause of recurrence even after radical glioma resection [4]. The process of tumor metastasis is generally considered to be divided into the following four steps: (1) separating of invading cells from the primary site; (2) adhesion to the extracellular matrix: (3) degradation of the extracellular matrix; (4) movement of the invasive cells [5, 6]. In addition, the heterogeneity of the cells within the tumor also promotes the metastatic capacity of the tumor cells. However, specific molecular mechanism of the metastasis of glioma is still unclear.

Traditional high-throughput sequencing data analysis is difficult to analyze the heterogeneity inside the tumor [7–9], while the single-cell sequencing technology developed in recent years allows researchers to view the expression profile of each cell and explore the expression heterogeneity of single cells within the tumor mass [10–12]. The discovery of intracellular difference provides the potential for a deeper understanding of tumor internal characteristics [13]. The purpose of this study is to analyze the glioma single-cell sequencing data, analyze the characteristics and marker genes of metastatic glioma cells, and analyze the prognostic value of these metastasis-associated genes (MAGs) with the help of high-throughput sequencing, thereby providing new targets for basic and clinical research for gliomas. Since IDH wild-type glioma has a worse prognosis than their mutant counterparts and shows more malignant biological behaviors [14, 15], IDH wild-type glioma were selected for study in order to better explore the metastatic characteristics of glioma.

## Methods

### Acquisition and quality control of glioma expression sequencing data

The glioma single-cell sequencing data were sourced from GSE84465 of the GEO database (Gene Expression Omnibus, https://www.ncbi.nlm.nih.gov/geo/). “limma” package of R software was used for preliminary data correction. “Seurat” package was used to analyze single-cell sequencing data. Genes expressed in less than 3 cells and cells expressing less than 50 genes were excluded. Percentage of mitochondrial sequencing count < 0.05 and the correlation between gene numbers and sequencing depth > 0.4 were used as the threshold to identifying high-quality single-cell sequencing samples. The high-throughput sequencing data of IDH wild-type glioma and patient clinical information were derived from the TCGA database (https://cancergenome.nih.gov/) and CGGA database (http://www.cgga.org.cn/). Since all the original data were downloaded from public databases, no additional ethical proof was required.

### Dimensionality reduction and clustering

In this study, the single-cell sequencing data was firstly subjected to dimensionality reduction using the principal component analysis (PCA) method. The t-Distributed Stochastic Neighbor Embedding (tSNE) analysis were then introduced based on the *P* values of each principal component. According to differentially expressed genes of each cluster by tSNE clustering and marker genes of brain tissues reported by previous articles, cells from each cluster were annotated.

### Gene set enrichment analysis (GSEA)

The “clusterProfiler” package of R software was used to analyze the MAGs in glioma by Gene Ontology (GO) and Kyoto Encyclopedia of Genes and Genomes (KEGG) enrichment analyses, and to explore the potential biological functions of these genes.

### Identification of MAGs and prognostic correlation

According to the cell annotations, differentially expressed genes of metastatic glioma cells and nonmetastatic glioma cells were identified. Heat-map and volcano-map of MAGs were drawn accordingly. In order to study the prognostic correlation of these MAGs, we extracted the expression profiles of these MAGs and patient survival information from glioma high-throughput sequencing data. Because glioma patients in TCGA database included normal brain tissue samples, we used TCGA database as the training cohort while CGGA database as the validation cohort. Firstly, univariate Cox regression was used to analyze the prognostic genes in the training cohort. Then multivariate Cox regression by Efron approximation was used to establish the risk score, which was expressed as: risk score = β_gene1_ × Expression_gene1_ + β_gene2_ × Expression_gene2_ + β_gene3_ × Expression_gene3_ + ... + β_genen_ × Expression_genen_. Using this model, the ROC curve (Receiver operating characteristic) and Kaplan-Meier survival curve were drawn in both the training cohort and the validation cohort respectively. The sensitivity and specificity of the model for the prognosis of glioma were analyzed.

## Results

### Quality control of single-cell sequencing profiles

**Fig. 1:**
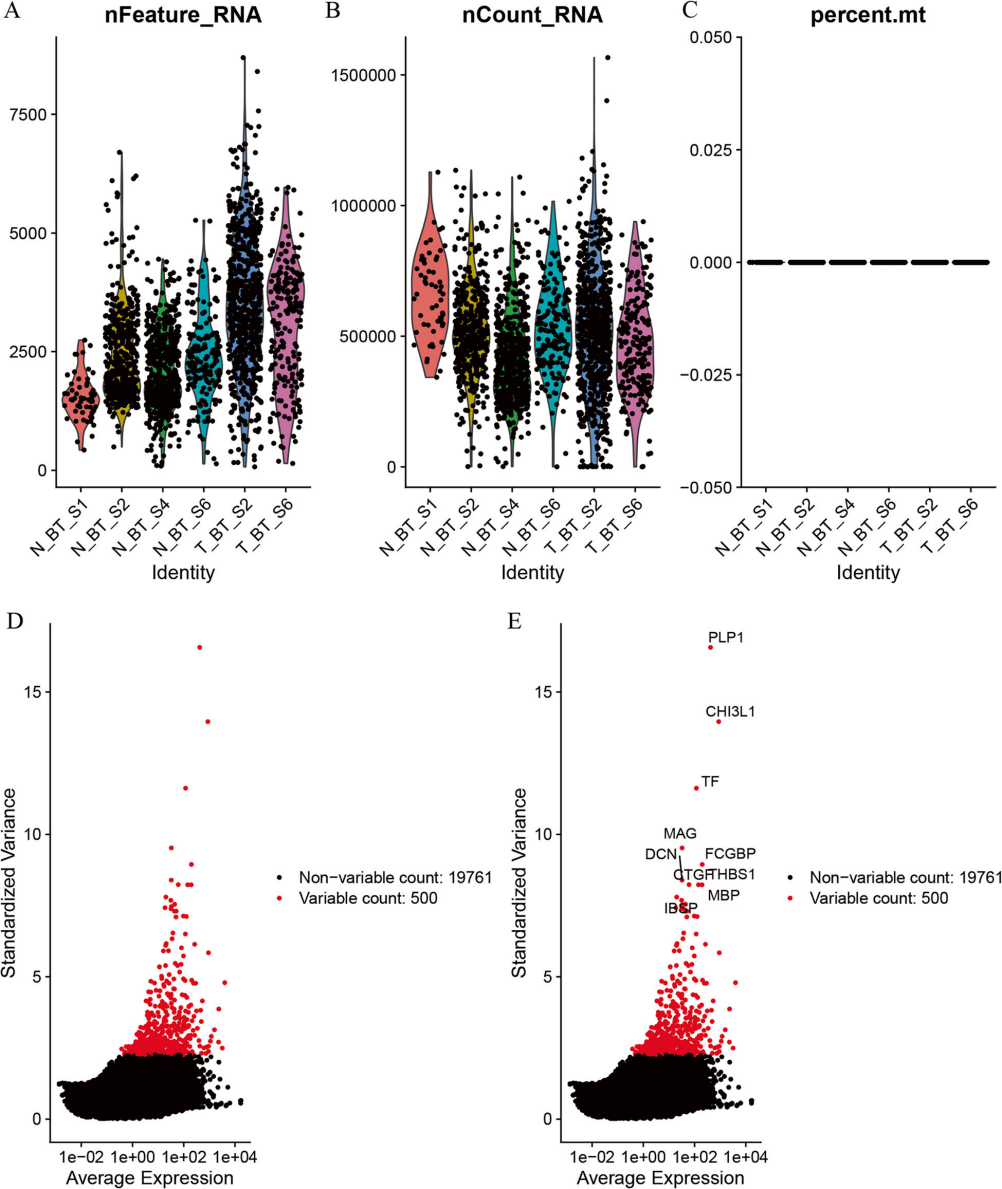
Overview of single-cell sequencing profiles. **a** The description of feature genes of the profiles. **b** The description of total gene numbers of the profiles. **c** Percentage of mitochondrial genes detected in each sample. **d**-**e** Diagrams of differentially expressed genes between cells

A total of 3589 cell expression data were obtained from the 4 tumor tissues and 4 surrounding peritumor tissues from the GSE84465. The number of gene types and total number of genes expressed in each sample and the correlation between the number of sequenced genes and the depth of sequencing in each sample were shown in Fig. S1. With Pearson correlation greater than 0.4 as the threshold, 4 surrounding peritumor tissues and 2 tumor samples (N_BT_S1, T_BT_S2, N_BT_S2, N_BT_S4, T_BT_S6, and N_BT_S6) were screened. These 6 eligible samples were used for subsequent analysis. The number of gene types and total number of genes expressed in the eligible samples were shown in Fig. 1a-b. In addition, all the percentages of mitochondrial sequencing count of samples were very low (Fig. 1c), which may be due to the single cell sorting process by antibodies before performing single-cell sequencing, resulting in very few apoptosis or lysed cells. Figure 1d-e showed the distribution of the 500 genes with the most intercellular expression differences and the names of the top 10 differentially expressed genes (including PLP1, CHI3L1, TF, MAG, FCGBP, DCN, CTGF, THBS1, MBP and IBSP).

### Clustering and cell annotation

**Fig. 2:**
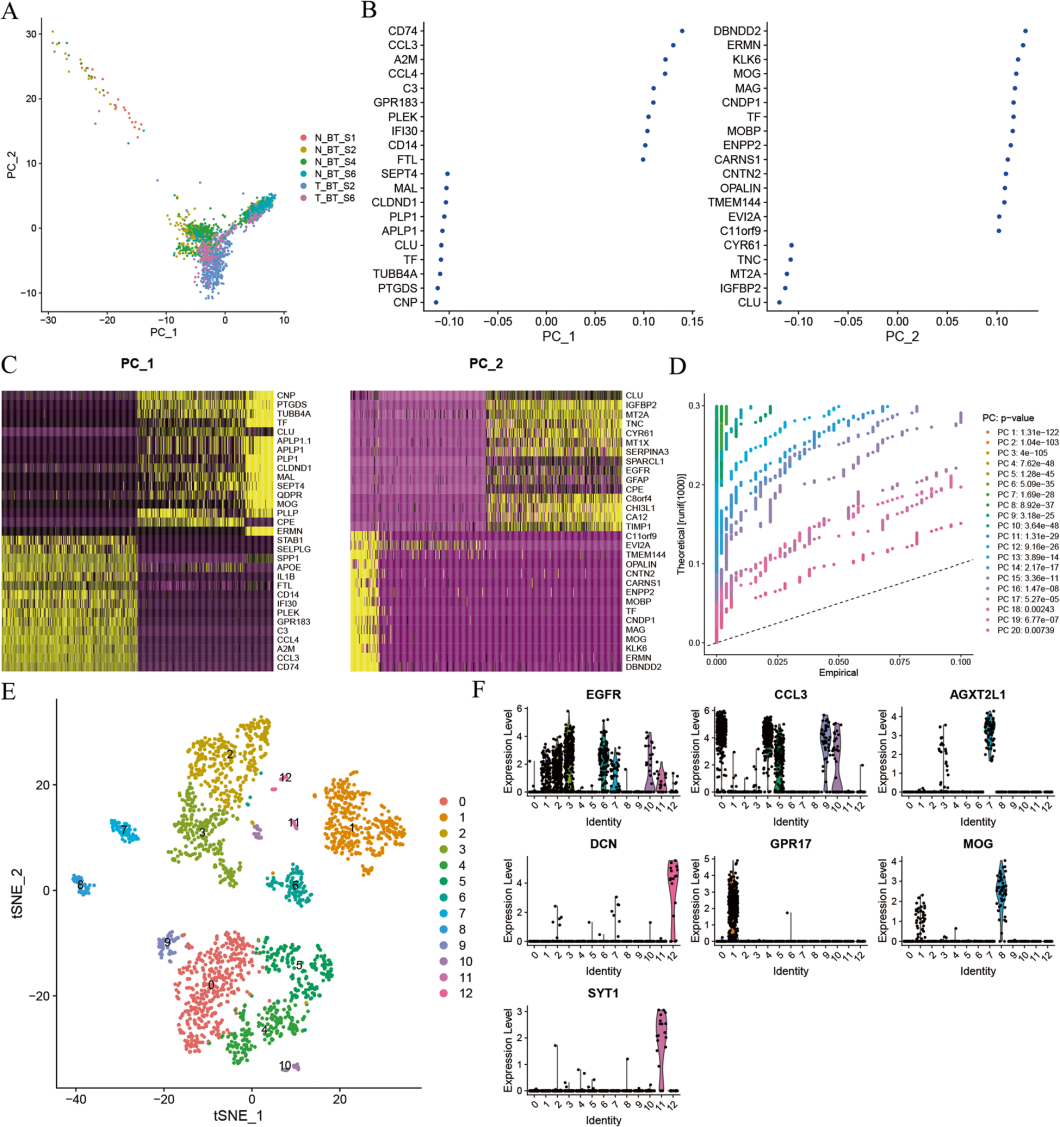
Clustering and cell annotation. **a** PCA results of single-cell sequencing profiles. **b** Top 20 principal genes of PC_1 and PC_2 clusters. **c** Heat-maps of PC_1 and PC_2 clusters. **d** *P* value of each PCA component. **e** tSNE results of single-cell sequencing profiles. **f** Expression of classical genes in various clusters

The PCA analysis results of single-cell sequencing data were shown in Fig. 2a. The glioma cells were differentiated clearly by the PC_1 and PC_2 clusters. In addition, Fig. 2b-c listed top 20 principal genes and corresponding heat-maps of PC_1 and PC_2 clusters. *P* value of each principal component was calculated, as shown in Fig. 2d. Significant principal components were further introduced to the tSNE dimensionality reduction in order to make glioma cells better clustered. The results of tSNE dimensionality reduction were shown in Fig. 2e. From the figure, we found that glioma cells were divided into 13 clusters named 0–12, and the glioma cells were more clearly distinguished than the PCA method. Among them, cluster 6 was only derived from tumor tissues, and cluster 7 and cluster 11 were only derived from surrounding peritumor brain tissue. According to existing literature reports [16–18], we analyzed the expression of marker genes of different brain tissues in various clusters (as shown in Fig. 2f). First, cluster 7 and 11 were adopted only from peritumoral brain tissue, indicating that they were not tumor cells. Cell-specific genes provide more important proofs for cell annotation. Meanwhile, Ye Zhang’s suggests that CCL3 can be the cell-specific gene for microglia/macrophages, AGXT2L1 for astrocytes, and SYT1 for neurons [18]. In addition, Spyros Darmanis points out in their study that endothelium-derived cells tend to express DCN, OPC cells tend to express GPR17, and oligodendrocytes tend to express MOG. More importantly, EGFR can discriminate glioma cells with high sensitivity and specificity [16]. From the above, cells belonging to cluster 2,3,6 and derived from peritumor brain tissue were defined as metastatic tumor cells. The differentially expressed genes between metastatic tumor cells and non-metastatic tumor cells were identified.

### Gene set enrichment analysis

**Fig. 3:**
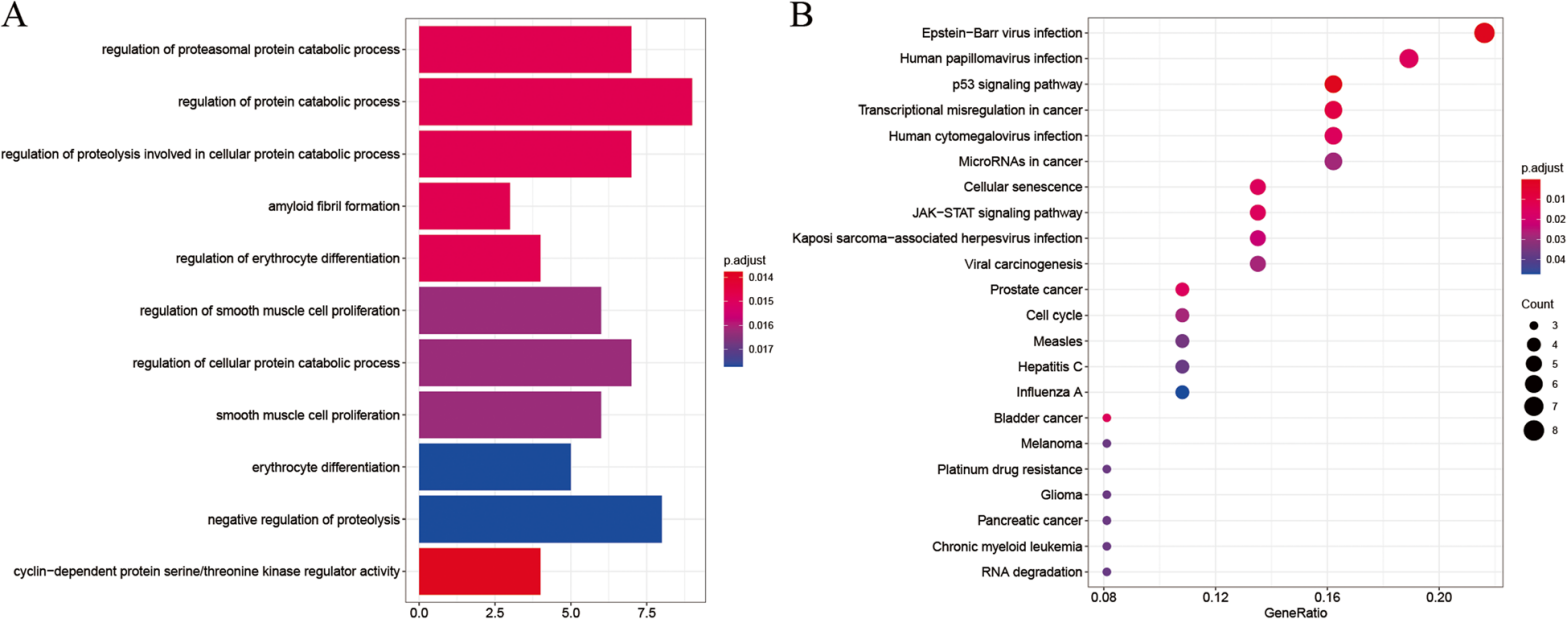
Gene set enrichment analysis of the MAGs. **a** GO enrichment results. **b** KEGG enrichment results

The expression and survival information were extracted from IDH wild-type glioma high-throughput sequencing data from the TCGA and CGGA database. The shared genes of TCGA and CGGA database were extracted for subsequent analysis. Wilcox test was applied to find the differentially expressed genes between IDH wild-type glioma and normal brain tissue. The intersection differentially expressed genes in IDH wild-type glioma and metastatic cells were defined as MAGs. The MAGs were analyzed by GO and KEGG enrichment analysis for their potential functions. The results were shown in Fig. 3. With *P* < 0.05 as the statistical standard, GO enrichment analysis suggested that genes were enriched on items such as regulation of protein catabolic process, regulation of proteolysis involved in cellular protein catabolic process. Meanwhile, KEGG enrichment analysis suggested that these genes may be involved in signal pathways such as p53 signaling pathway, transcriptional misregulation in cancer, JAK-STAT signaling pathway.

### Construction of prognostic model based on MAGs

**Fig. 4:**
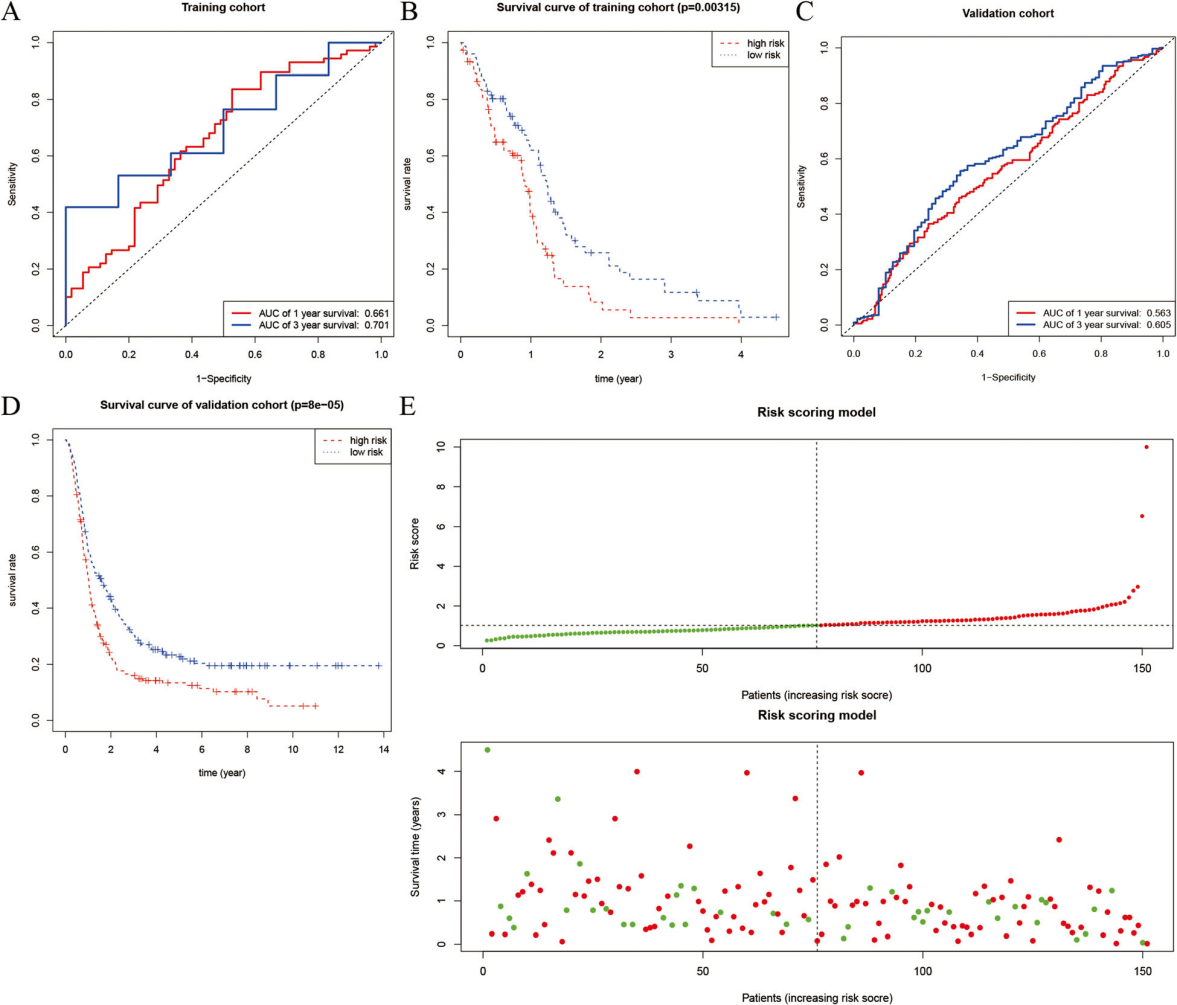
Cox regression. **a** 1-year and 3-year ROC curves in the training cohort. **b** Kaplan–Meier survival analysis between high-risk group and low-risk group in the training cohort. **c** 1-year and 3-year ROC curves in the validation cohort. **d** Kaplan–Meier survival analysis between high-risk group and low-risk group in the validation cohort. **e** The relationship between the risk scores and overall survival

Univariate Cox regulation was used to analyze the MAGs to find out the prognostic genes and 79 MAGs were associated with overall survival of IDH wild-type glioma patients. Using the multivariate Cox regression in the training cohort, a total of 3 MAGs were identified for constructing the prognostic model, including GNS (glucosamine (N-acetyl)-6-sulfatase), LBH (LBH regulator of WNT signaling pathway) and SCARA3 (scavenger receptor class A member 3). The Cox results of these genes were shown in Table 1. Risk score = 0.516 * Expression _GNS_ + 0.422 * Expression _LBH_ + 0.211 * Expression _SCARA3_. The value for Likelihood ratio test was 19.84 (*P* < 0.001), 18.94 for Wald test (*P* < 0.001) and 18.95 for Score (logrank) test (*P* < 0.001). Based on the risk score calculated by the prognostic model, the ROC curves and Kaplan-Meier survival curves for the training and validation cohorts were drawn in Fig. 4a-d.

**Table 1:** Univariate analysis and multivariate Cox results of metastasis-associated genes

RNA	Univariate Analysis		Multivariate Analysis	
	HR (95%CI)	***P***	HR (95%CI)	***P***
GNS	1.661 (1.161–2.378)	**0.006**	1.676 (1.131–2.482)	**0.010**
LBH	1.499 (1.118–2.010)	**0.007**	1.524 (1.117–2.081)	**0.008**
SCARA3	1.146 (1.119–1.791)	**0.004**	1.234 (0.956–1.593)	0.106

The results showed that the AUCs (the area under ROC curves) for 1-year and 3-years OS (overall survival) of the training cohort were 0.661 and 0.701, respectively; the AUC for the 3-year and 5-year OS of the validation cohort were 0.563 and 0.605, respectively. In addition, the *P* values of Kaplan-Meier survival curves of the training and validation cohorts were 0.003 and 0.000 respectively. The prognostic model based on MAGs showed an excellent prognostic ability for IDH wild-type glioma. Furthermore, the relationship between the risk scores and overall survival were shown in Fig. 4e.

## Discussion

The metastasis is an important reason for the difficulty of glioma treatment. This study combined single-cell sequencing and high-throughput sequencing to study the characteristics of MAGs of IDH wild-type glioma. First, through quality control, we selected six eligible single-cell sequencing profiles for further research. Second, the PCA and tSNE dimensionality reduction were used to classify all cells into 13 clusters according to the differentially expressed genes between cells. According to the cell annotation results, cluster 2, cluster 3 and cluster 6 were identified as glioma cells. A small percentage of cells in cluster 2 and cluster 3 originated from surrounding peritumor tissue, and theses cell were defined as metastatic cells. MAGs of glioma were gained by comparison between metastatic cells and non-metastatic cells.

Due to the small number of patients with single-cell sequencing, it is difficult to complete survival analyses of MAGs. Therefore, we extracted high-throughput sequencing data of glioma from the TCGA database for prognostic analyses of MAGs. GSEA results suggested that these MAGs might be involved in signal pathways such as regulation of proteolysis involved in cellular protein catabolic process (CLU/HSP90AB3P/MDM2/OS9/RNF180/SDCBP/TRIB2), p53 signaling pathway (CASP3/CCND2/CDK4/CDKN1A/IGFBP3/MDM2), transcriptional misregulation in cancer (CCND2/CDKN1A/IGFBP3/MDM2/PLAT/ZEB1) and JAK-STAT signaling pathway (CCND2/CDKN1A/FHL1/GFAP/STAT1). For example, MDM2, a negative regulator of P53, inhibits P53 transcription and promotes ubiquitination and degradation of P53 through proteasome [19]. CDKN1A, whose gene promoter region contains TP53 binding sites, can be a mediator of p53 signaling pathway [20]. Moreover, CCND2 is a cyclin expressed mainly in glioma stem cells. Knockdown of CCND2 can decrease levels of E2F1, E2F2 and cyclin B1, leading to a significant increase in the proportion of G1 phase cells [21]. In addition, STAT1 has an antagonistic effect on cellular proliferation and apoptosis through the JAK-STAT signaling pathway [22]. Interestingly, STAT3, also a member of the STAT protein family, has been found in many studies to be phosphorylated and mediate drug resistance in cancer therapy [23, 24]. With *P* < 0.05 as the statistical threshold, STAT3 was highly expressed in metastatic glioma cells, which also indicated the accuracy and application prospect of single-cell sequencing research.

Through Cox regression, this study selected 3 MAGs (GNS, LBH and SCARA3) to construct the prognostic model. The ROC curves and Kaplan-Meier survival curves of the training cohort and the validation cohort indicated that the model had good prognostic ability.

This study identified the MAGs of IDH wild-type glioma and the prognostic model was constructed by genes including GNS, LBH and SCARA3. Many of these genes have been reported in previous studies. For example, LBH is highly expressed in glioma. Under hypoxic conditions, LBH is directly regulated by HIF-1, and promotes glioma angiogenesis in human brain microvessel endothelial cells through the VEGFA-mediated ERK signaling pathway [25]. SCARA3 mRNA is highly expressed in breast cancer [26]. Besides, SCARA3 promotes drug resistance in multiple myeloma [27]. The mechanism of more metastasis-associated genes of glioma remains to be discovered.

Studies on the analyses of RNA sequencing and prediction of the prognosis of IDH wild-type glioblastoma have been reported [28]. Since metastasis is generally considered as a pivotal factor of prognosis of glioma, the innovation of this paper is to identify the molecular characteristics of metastatic glioma cells by analyzing single-cell sequencing of glioma, and to use high-throughput sequencing for prognostic validation. The study may help provide deeper insight into genes involved in glioma cell metastasis.

This study also has some shortcomings, such as lacking of overexpression/deletion studies and validation in vivo experiments which may further examine the function and mechanism of candidate genes.

## Conclusions

In conclusion, this study explored glioma MAGs through single-cell sequencing data mining, and studied the prognostic value of these genes for glioma patients. The aim of this study is to identify prognostic metastasis-associated signatures for glioma and may provide potential targets for further cancer research.

## Supplementary Information

**Additional file 1 **: **Figure S1**. The correlation between the number of sequenced genes and the percentage of mitochondrial genes, and the correlation between the number of sequenced genes and the depth of sequencing in each sample. (A: T_BT_S1; B: N_BT_S1; C: T_BT_S2; D: N_BT_S2; E: T_BT_S4; F: N_BT_S4; G: T_BT_S6; H: N_BT_S6).

## Data Availability

All original data in the manuscript are available upon reasonable request.

## Abbreviations

MAGs: Metastasis-associated genes
OS: Overall survival
LASSO: Least absolute shrinkage and selection operator
PCA: Principal component analysis
tSNE: t-Distributed Stochastic Neighbor Embedding
logFC: Logarithm of fold change
adjPval: Adjustment of *P* value
ROC curve: Receiver operating characteristic
AUC: The area under ROC curves
GSEA: Gene set enrichment analysis
GO: Gene Ontology
KEGG: Kyoto Encyclopedia of Genes and Genomes
