# Supplementary figures and images for: Analyses of metastasis-associated genes in IDH wild-type glioma
Source: BMC Cancer. 2020 Nov 16;20:1114. doi: 10.1186/s12885-020-07628-0 (PMC7670782; doi:10.1186/s12885-020-07628-0)

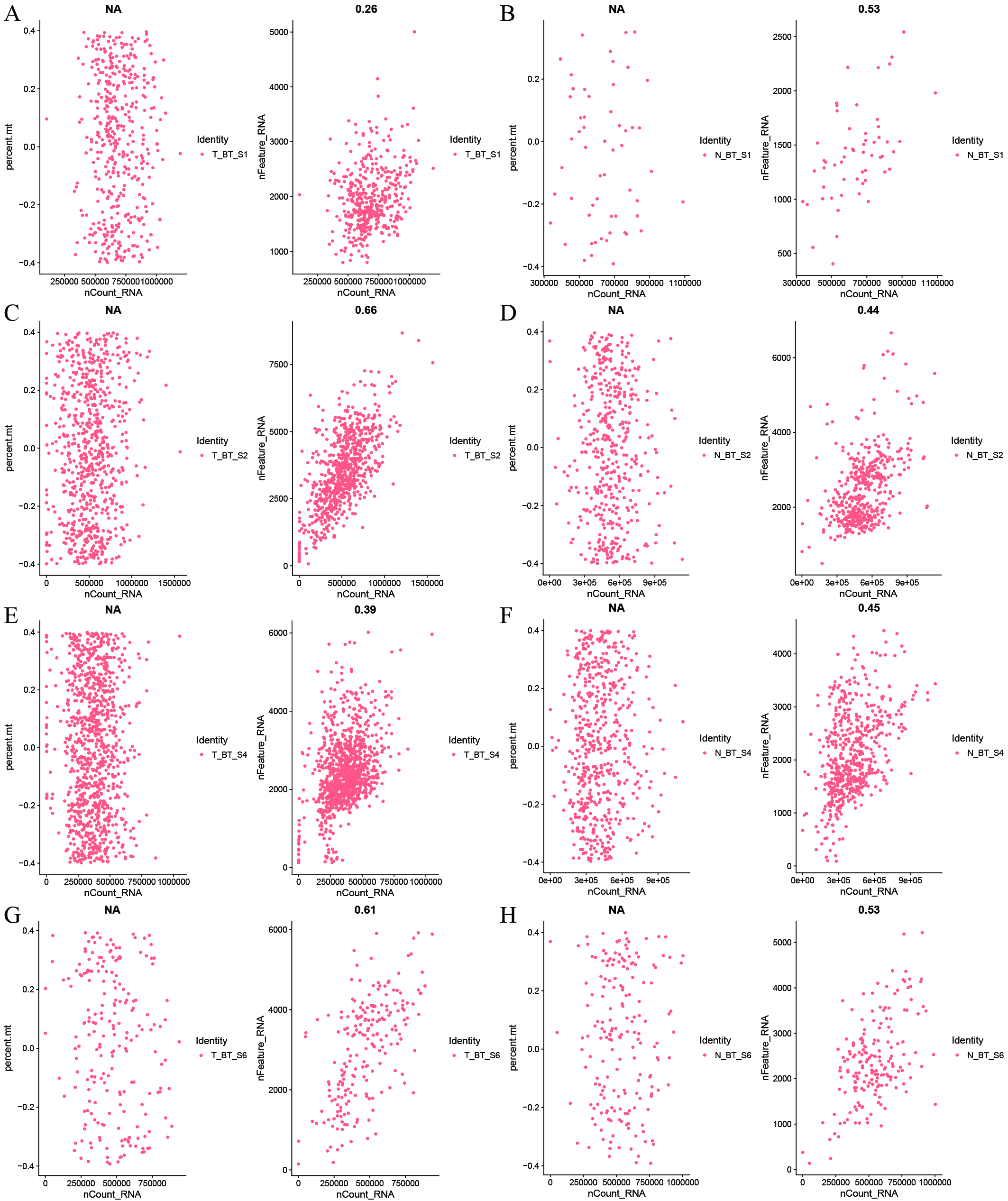

Supplement: Supplementary file 1 — Additional file 1 : Figure S1. The correlation between the number of sequenced genes and the percentage of mitochondrial genes, and the correlation between the number of sequenced genes and the depth of sequencing in each sample. (A: T_BT_S1; B: N_BT_S1; C: T_BT_S2; D: N_BT_S2; E: T_BT_S4; F: N_BT_S4; G: T_BT_S6; H: N_BT_S6). [file 12885_2020_7628_MOESM1_ESM.tif]
